# O061: Impact of process control (PC) implementation and strategies to improve hand hygiene adherence (HHA), in device-associated infections (DAI) in an intensive care unit of adults (AICU)

**DOI:** 10.1186/2047-2994-2-S1-O61

**Published:** 2013-06-20

**Authors:** C Giuffre, ED Efrón, AM Azario, R Jordan, JV Martinez, S Verbanaz, P Giorgio, M Khoury

**Affiliations:** 1 Infection Diseases, Buenos Aires British Hospital, Buenos Aires, Argentina; 2 Research & Teaching, Buenos Aires British Hospital, Buenos Aires, Argentina

## Introduction

DAI are a serious public health problem worldwide. The PC and optimization of the HHA have proven to be excellent tools for DAI minimization or elimination.

## Objectives

Assess the impact of PC and HHA on DAI in our AICU.

## Methods

A quasi-experimental study. We calculated the average rates of DAI using NHSN,CDC methodology in two periods:pre (Pa) and post intervention (Pb). Average rates wereexpressed as number of events per 1000 device days (DD). Study periods: ventilator associated pneumonia (VAP) Pa: January 2004-January 2010, Pb: February 2010-February 2013. Catheter-associated bacteremia (BACT) Pa: January 2004-June 2010, Pb: July 2010-February 2013. Catheter-associated urinary tract infection (UTI) Pa: January 2007-October 2011Pb: November 2011-February 2013. The Institute of Healthcare Improvement (IHI) proposed PC were implemented. PC adherence was assessed periodically. In October 2009, WHO campaign to improve HHA was implemented. Periodic measurements of HHA were performed. Statistical analysis: we compared average rates of HAI in both periods with Mann-Whitney test. We calculated the Incidence Rate Ratio (IRR) as Pb average incidence rate/Pa average incidence rate.

## Results

NEU rate decreased from 9.88 (Pa) to 2.60 (Pb), P <0.001. IRR 0.26, 74% rate reduction. Attributable risk: 7.28/ 1000 DD. Cases avoided in Pb: 33.4 (4589 DD in three years). BACT rate decreased from 5.35 (Pa) to 2.34 (Pb), p 0.007. IRR: 0.44, 56% rate reduction. Attributable risk: 3.01/ 1000 DD. Cases avoided in Pb: 14.99 (4983 DD in 32 months). ITU rate decreased from 2.45 (Pa) to 1.30 (Pb), P 0.32. IRR: 0.53, 47% rate reduction. Attributable risk: 1.15/ 1000 DD. Cases avoided in Pb: 3.34 (2908 DD in 16 months). Adherence to PC and HHA ranged between 80 and 95%.

## Conclusion

PC implementation and HHA optimization in our AICU was associated with statistically significant decreases in VAP and BACT rates, and similar tendency of UTI rate. The significant number of cases averted, fully justifies the implementation of these tools.

## Disclosure of interest

None declared.

